# Physical Activity, Sleep, and Nutrition Do Not Predict Cognitive Performance in Young and Middle-Aged Adults

**DOI:** 10.3389/fpsyg.2016.00642

**Published:** 2016-05-03

**Authors:** Hieronymus J. M. Gijselaers, Barberà Elena, Paul A. Kirschner, Renate H. M. de Groot

**Affiliations:** ^1^Faculty of Psychology and Educational Sciences, Welten Institute—Research Centre for Learning, Teaching and Technology, Open University of the NetherlandsHeerlen, Netherlands; ^2^eLearn Center, Universitat Oberta de CatalunyaBarcelona, Spain; ^3^Department of Complex Genetics, Faculty of Health, Medicine and Life Sciences, School for Nutrition, Toxicology and Metabolism, Maastricht UniversityMaastricht, Netherlands

**Keywords:** biological lifestyle factors, sedentary behavior, the ALOUD study, path analysis, structural equation modeling, trail making test, N-back task, substitution test

## Abstract

Biological lifestyle factors (BLFs) such as physical activity, sleep, and nutrition play a role in cognitive functioning. Research concerning the relation between BLFs and cognitive performance is scarce however, especially in young and middle-aged adults. Research has not yet focused on a multidisciplinary approach with respect to this relation in the abovementioned population, where lifestyle habits are more stable. The aim of this study was to examine the contribution of these BLFs to cognitive performance. Path analysis was conducted in an observational study in which 1131 adults were analyzed using a cross-validation approach. Participants provided information on physical activity, sedentary behavior, chronotype, sleep duration, sleep quality, and the consumption of breakfast, fish, and caffeine via a survey. Their cognitive performance was measured using objective digital cognitive tests. *Exploration* yielded a predictive cohesive model that fitted the data properly, χ^2^*/df* = 0.8, CFI = 1.00, RMSEA < 0.001, SRMR = 0.016. *Validation* of the developed model indicated that the model fitted the data satisfactorily, χ^2^*/df* = 2.75, CFI = 0.95, RMSEA < 0.056, SRMR = 0.035. None of the variables within the BLFs were predictive for any of the cognitive performance measures, except for sedentary behavior. Although sedentary behavior was positively predictive for processing speed its contribution was small and unclear. The results indicate that the variables within the BLFs do not predict cognitive performance in young and middle-aged adults.

## Introduction

Cognitive performance is influenced, amongst other things, by physical activity (e.g., active vs. passive behavior; Burkhalter and Hillman, 2011; Voss et al., 2011), sleep (e.g., sleep duration and quality; Dean et al., 2010; Philip et al., 2012), and nutrition (e.g., breakfast and poly-unsaturated fatty acids; Burkhalter and Hillman, 2011). A comprehensive perspective of behavior within these three biological lifestyle factors (BLFs) and their relation with cognitive performance in adults is needed for a number of reasons. Healthy behavior concerning these controllable environmental factors has been proven to result in better physical and mental health (Busch et al., 2013), but also leads to better cognitive performance (Small et al., 2006). Previous research has focused mainly on children and adolescents however, and solely on separate domains or even variables within these domains (e.g., only chronotype: Vollmer et al., 2013). To our knowledge there is no research focusing on the combination of these three BLFs and the relation with cognitive performance in young and middle-aged adults, an age group in which lifestyle habits are more stable in comparison to their younger counterparts. Therefore, the goal of the current study was to evaluate whether subjectively measured general habitual behavior on the three BLFs physical activity, sleep, and nutrition was predictive for cognitive performance in young and middle-aged adults using path analysis.

Physical activity, sleep, and nutrition all exert influence on the regulation of the body's physiological parameters. For example, the levels of brain-derived neurotropic factor (BDNF) increase when being physically active (Winter et al., 2007), hereby enhancing the proliferation, synaptic plasticity, growth and survival of neurons (van Praag, 2009). Melatonin, a hormone produced by the pineal gland, regulates sleep onset. Later bed times in these contemporary times means more artificial light exposure later at night, shifting the melatonin onset (Wright et al., 2013). n–3 long chain poly-unsaturated fatty acids (n-3 LCPUFAs), such as omega-3 found in foods like fish and nuts, are a good example of nutritional elements influencing the body. n-3 LCPUFAs are important building blocks for cell membranes, providing the proper permeability and fluidity which is important for signal transduction in neurons (Gómez-Pinilla, 2008). In addition, they also have a direct impact on BDNF levels (i.e., omega-3; van Praag, 2009).

Many of the physiological mechanisms influenced by physical activity, sleep, and nutrition have implications for brain functioning and thus possibly also on cognitive performance. The goal of this paper is not to provide a review of all possible mechanisms related to physical activity, sleep, and nutrition and their effect on brain functioning. Readers that would like an overview of the possible mechanisms can refer to the following sources of literature for physical activity (Barenberg et al., 2011), sleep (Tononi and Cirelli, 2014), and nutrition (Gómez-Pinilla, 2008).

Cognition is a term that is often used to refer to the mental abilities that facilitate processes such as memory, planning, inhibition, and problem-solving. These processes range from simple lower-order processes such as processing speed, to more complex higher-order processes such as task switching. In the cognitive domain, the executive functions (EFs) are considered to be very important for normal adult performance (Salthouse et al., 2003). EFs are top-down controlled mental processes that are needed for concentration and attention; the use of which take effort. When EFs are not used this implies that individuals act habitually, do not change their ways, and give in to temptations (Diamond, 2013). Despite the many definitions for EFs and the components that belong to them, the former description is generally agreed upon (Jurado and Rosselli, 2007). This review provides an in-depth examination of the concept, its components, related brain areas, and related tests (Jurado and Rosselli, 2007). In essence, some researchers believe that EFs have a unifying, central factor (Duncan et al., 1996; de Frias et al., 2006), while other believe that EFs depend on separate processes (Miyake et al., 2000; Salthouse, 2005). It remains to be determined which underlying processes relate to all EFs. For the data gathered and the tests performed in this study, Miyake et al. (2000) provide the best model as compared to others (Fisk and Sharp, 2004; Salthouse, 2005). The three EFs described by this model are often mentioned in literature, all of which are based on Baddeley's model of working memory (Baddeley, 1983) and his later proposal on the functions performed by the “central executive” (Baddeley, 1996). Miyake et al. (2000) described these functions as inhibition, updating, and shifting after statistically having analyzed Baddeley's proposal. To clarify, working memory can be divided into two components, short-term storage and executive processes (Smith and Jonides, 1999). The executive function “updating” manipulates the short-term storage and together with the “storage” forms the working memory.

Physical activity is defined as any movement originating from skeletal muscles that requires energy expenditure and has various subcategories (e.g., exercise or work-related activity) and indicators (e.g., subjective or objective). There is a large amount of research focusing on the positive effects of physical activity on cognitive processes (e.g., the reviews of Colcombe and Kramer, 2003; Hillman et al., 2008; Barenberg et al., 2011). The *executive function hypothesis* proposed a preferential benefit of physical activity for EFs (Hall et al., 2001). Indeed, such differential effects of physical activity on different cognitive functions exist. EFs benefit most from physical activity, while processing speed seems to benefit least (Colcombe and Kramer, 2003). In their extensive review article, Barenberg et al. (2011) discussed the limitations of the study of Colcombe and Kramer (2003) and investigated whether this preferential benefit of physical activity for EFs was apparent. They concluded that the results point in the direction of a preferential benefit (Barenberg et al., 2011). Important to note is that, according to the three EFs (i.e., updating, shifting, inhibition) as defined by Miyake et al. (2000), updating has never been investigated, consistent positive effects were shown on inhibition, and in the shifting domain positive effects were found occasionally (Barenberg et al., 2011). These research findings focus on direct effects of physical activity and not on overall habitual physical activity behavior. This is important as measures within the construct physical activity are diverse and different measures can be differentially associated with cognitive performance (e.g., Syväoja et al., 2014). In this study we focus on general habitual physical activity and on overall behavior as an indicator of physical activity.

Traditionally seen as part of physical activity, sedentary behavior refers to any behavior in which someone is using >1.5 metabolic equivalents. Generally this occurs when an individual is either sitting or lying down. In recent research the construct sedentary behavior is more often viewed as a separate construct, independent of physical activity, as a large review study found that general sedentary behavior is not related to physical activity (Rhodes et al., 2012). This is not surprising as one can be highly physically active and still sit a large amount of the day, due to a sedentary job, for example, which is a very likely situation in these contemporary times. Furthermore, it is important to evaluate whether this independence also exists with respect to cognitive performance. Research regarding sedentary behavior and its relation to cognitive performance is still very scarce, especially in young and middle-aged adults. There are some signs that lower amounts of sedentary behavior in combination with low-intensity physical activity behavior could be counteractive in age-related cognitive decline and that biological mechanisms underlying sedentary behavior and physical activity could overlap and possibly counteract their effects (Voss et al., 2014). To specify, these signs include the overall activity relation with cognitive performance in which moderate-to-vigorous physical activity barely accounted for the age-related cognitive decline (Smith, 2012).

In children (Dewald et al., 2010), adolescents (Radek and Kaprelian, 2013), and older adults (Nebes et al., 2009) sleep deprivation, due to low sleep quality or simply too little sleep, has been proven to lead to impaired cognitive performance. Besides actual sleep, chronotype also plays a role in cognitive performance. Chronotype, or time-of-day preference, refers to a person's preference for mornings or evenings (for more information, see Roenneberg et al., 2003). As shown in a meta-analytic study, evening oriented people generally perform better than morning oriented people, independent of the time at which the cognitive tests were performed (Preckel et al., 2011). In adults, ideal sleep duration is 7–8 h per night. Shorter as well as longer sleep duration leads to lower cognitive performance (Ferrie et al., 2011; Sternberg et al., 2013). Although sleep quality is a heavily researched domain, research concerning cognitive performance in adults, and in particular middle-aged adults, is scarce. Research in young adults shows poor sleep quality to be associated with decreased executive performance, specifically shifting (Benitez and Gunstad, 2012). In older adults, poor sleep quality has been associated with lower cognitive functioning. These relations were not uniform however across different cognitive functions. Impaired sleep quality has also been associated with decreases in working memory and shifting, but not in processing speed (Nebes et al., 2009). Both these findings align and show that higher order executive functions appear to be more affected by sleep quality than lower order functions.

We will evaluate the nutritional components that were measured in this study: breakfast, fish, and caffeine consumption. Breakfast consumption has been heavily investigated, albeit mainly in children. No generally accepted definition of breakfast is apparent in previous research and definitions range from “the first meal of the day” to “what participants themselves consider breakfast” (Mullan and Singh, 2010). Breakfast, as its name suggests, is mostly viewed as giving the body energy after a night of fasting and effects on cognitive performance are therefore mostly viewed as direct. Theoretically however, consuming breakfast could also have long-term benefits as a result of increased nutrient intake and better nutritional status (Pollitt and Mathews, 1998). Breakfast is a meal that is often skipped (Mullan and Singh, 2010). The general belief that breakfast is “the most important meal of the day” seems however to be far-fetched as this review shows that there is no general “recipe” for breakfast in relation to cognitive performance (Zilberter and Zilberter, 2013). This opinion article describes how seven distinct breakfast types have 16 different cognitive effects on nine populations.

Although research shows that n-3 LCPUFAs—found mainly in fish—are important for normal development, it is unknown whether n-3 LCPUFAs are beneficial for cognition in adults (Stonehouse, 2014). A major reason for this is simply because little research has been done in healthy young and middle-aged adults. Most research focuses on the development of children, adolescents, and older healthy or demented adults (Luchtman and Song, 2013). Research results in these age groups indicate that cognitive performance is enhanced following supplementation of n-3 LCPUFAs, although more research is needed to confirm this (McCann and Ames, 2005; Frensham et al., 2012; Stonehouse, 2014).

Caffeine can be regarded as a nutrient, a drug or a drug of abuse, depending on the way it is used (Pardo Lozano et al., 2007). Since we investigated habitual caffeine use in a healthy adult population, we considered caffeine as a nutrient. Caffeine is known to boost various cognitive functions and habitual caffeine intake is related to better long-term memory (Hameleers et al., 2000), alertness (Owen et al., 2008), reaction time, and short term recall (Ruxton, 2008). It is disputed however whether habitual intake truly enhances cognition and is not just a result of reversal of the withdrawal state. The *withdrawal reversal* hypothesis states that lower cognitive performance and alertness follows withdrawal and that this is restored by the consumption of caffeine, however no enhancement—separate from restoring the original performance—is apparent (Rogers, 2007). A recent review illustrates that evidence is in favor for the *withdrawal reversal* hypothesis (Rogers, 2014). Furthermore, consuming caffeine later in the night could also disrupt subsequent sleep processes which, as shown in adolescents (James et al., 2011), could impair cognitive performance.

In the present study, we examined whether variables within the BLFs physical activity, sleep, and nutrition were predictive for cognitive performance in young and middle-aged adults. We set out to investigate the joint contribution of subjectively measured general habitual behavior on these BLFs and expected that indicators of physical activity and sleep would be significant predictors of cognitive performance, while nutrition would not, following the literature review. The focus of this article will be on three cognitive functions: processing speed, shifting, and updating. As stated, more research is especially needed on the EFs shifting and updating (Nebes et al., 2009; Barenberg et al., 2011). In addition, as we measured cognitive performance in young and middle-aged adults, it is imperative to measure processing speed. First, because many cognitive processes are dependent on processing speed. Taking processing speed into account provides more interpretable information on the EFs. Second, aging causes cognitive processes to decline. This effect is independent and it is larger for processing speed than for EFs (Albinet et al., 2012). It is therefore important to account for processing speed as it can show insight in the unique age-related decline in each cognitive process.

The study was executed among young and middle-aged adult students of the Open University of the Netherlands (OUNL), a formal university-level institute providing distance education. As we had three outcome measures which were related to each other, path analysis using structural equation modeling was used. This method provides a measure of overall agreement, also called “fit,” between the model and the data as opposed to traditional path analysis using separate multiple regressions. As research in this adult population regarding BLFs and cognitive performance is scarce, a cross-validation approach was used with an *exploratory* and *confirmatory* mode to ensure validity of the analyses. The *exploratory* mode was used for model development, while the *confirmatory* mode was used for validation of the developed model.

## Materials and methods

### Design

Data from this observational study come from the Adult Learning Open University Determinants (ALOUD) study. The ALOUD study is an investigation of different psychological and biological factors possibly affecting cognitive performance and/or learning performance in students participating in distance education (Neroni et al., 2015). Data from the biological part of this project are available and stored permanently on DANS EASY, a sustainable platform for archiving research data (Gijselaers, 2015). All variables within the BLFs were reported via an online digital survey conducted after registration at the university. Cognitive performance was measured objectively using digital cognitive tests conducted via the participants' computer directly after the survey.

### Participants

Throughout 1 year (Sept. 2012–Aug. 2013), all new students of the OUNL who signed up for one or more regular bachelor or master course(s) were invited to participate. At the OUNL, students can register and start throughout the year as the education is modular and self-paced, open to everyone (with an age of at least 18 years old), and the curriculum is not fixed. This means that students can study full or part time. The OUNL mainly delivers online education.

The approached population size was 4945, 57.5% of those approached responded (*N* = 2842) and 41.27% of those approached (*N* = 2040) fully participated. From the sample of students that fully participated, the majority of students studied part time as most students had a full or part time paid job (i.e., 85.2%). Most students either lived alone (i.e., 20.4%), with a partner (i.e., 27.6%), or with partner and children (i.e., 34.3%). The age of participants ranged from 18 to 80, with the largest part (i.e., 56.9%) being between 26 and 45 years old. These participants are similar to the general population of students who normally study at the OUNL (Moerkerke, 2014).

### Procedures

Participants were invited automatically via the e-mail system of the university 14–21 days after successful registration. This 7 day range is because a bulk mailing was sent weekly. Students received a reminder 2 weeks after the initial invitation and 1 week later a last reminder via e-mail. Four weeks after the initial invitation, a phone call was made (the goal was to reach participants in the three subsequent weeks) in which potential participants were asked whether they were still interested in participating. If so, they received the original invitation once more when needed and a reminder 6.5 weeks after the initial invitation, which was around 1.5 weeks after the phone call. In case the phone call was made in week 6, the reminder was sent 1 week later. Participants only received reminders or a telephone call if no full response was recorded.

The survey was administered online using LimeSurvey®, version 1.92+ (LimeSurvey Project Team/Carsten, Schmitz, 2012). Full participation cost the participants 45–60 min on average and it was possible to stop and continue later, allowing participants more freedom in their participation by spreading the time burden. It was only possible to interrupt and continue later in between questionnaires. Most participants (i.e., more than 92%) concluded the cognitive tests in one go, as advised beforehand. If participants broke a task off before completing it, they had to do the complete task again. Students who fully participated could win (5% chance) a gift voucher of €20. The ALOUD study was ethically approved by the local ethical committee of the OUNL. Each participant signed a digital informed consent form, explicating the use of the personal data gathered, voluntary participation, possibility to withdraw at any time, and finally giving their permission to use the data for the described goals. Participants had to click a checkbox to agree with the terms mentioned; a mandatory action to start the survey.

### Materials

#### Outcome measures

Cognitive performance was measured by an online digital cognitive test battery which was collected after the survey. Three tests were administered in the following order: (1) the Trail Making Test (TMT) (Army Individual Test Battery, 1944); (2) the Substitution Test (ST), which resembles the symbol digit modalities test (Smith, 1991); (3) and the N-back task (NBT) (Lezak et al., 2004). The TMT consisted of four parts, namely two training sessions and two test sessions. The A-part which involved clicking randomly placed numbers as fast as possible in the correct order (i.e., 1, 2, 3, etc.), and the B-part which involved clicking randomly placed numbers and letters as fast as possible in the correct order in a shifting mode (i.e., 1, A, 2, B, 3, C, etc.). Both parts were preceded by an instruction and a practice session. The ST consisted of two parts, namely one training sessions and one test session. The participants had to match the symbol shown with the correct number from a key on the top of the page. After clicking any number, the next symbol came up. They were instructed to substitute as many items possible in 90 s. The NBT consisted of four parts; three training sessions and one test session. The participants performed a two-back task with 60 items in which they had to indicate whether the number shown was identical to the number shown two trials earlier. For all three tests the participants were instructed to work as accurately and quickly as possible. The TMT resulted in a measure for the executive function shifting, measured via the B-A part, in which the number part was subtracted from the number-letter part. The B-A part provides a relatively pure indicator of task-switching ability that minimizes for working memory and visuoperceptual demands (Sánchez-Cubillo et al., 2009). The outcome measure in the ST was the number of items correctly substituted in 90 s, which is a measure of processing speed. This ST mainly measures perceptual processing, visual search, and involves a motor component (e.g., Shum et al., 1990; Sánchez-Cubillo et al., 2009). In the NBT, the number of correctly remembered items is a measure for working memory and the executive function updating. Updating tasks, such as the NBT, measure general working memory processes as well as unique substitution processes which are independent of working memory (Ecker et al., 2010; Wilhelm et al., 2013).

#### Predictors

The measures for the variables within each BLF were extracted from various questionnaires. First the sleep related measures were questioned, then physical activity, and then nutrition. All BLF measures were related to habitual behavior over the past months. *Physical activity* was measured via the Short Questionnaire to ASsess Health-enhancing physical activity (SQUASH), which has a reasonable reliability (*r* = 0.58) and validity (*r* = 0.45; validated against an accelerometer) (Wendel-Vos et al., 2003). Participants were asked how many days a week they spent on the activities and how much time on an average day. Physical activity was calculated as a weekly activity score; an accumulated product score of intensity of the activity multiplied by the minutes spent on the activity. The activity score represented habitual physical activity on the basis of an average week over the past months. The questions included information regarding activities related to commuting, leisure time, household, and work. The leisure time activities were walking, cycling, gardening, and four sports to be filled in by the participants. The reported sports were manually inspected and rescored to corresponding metabolic equivalent values reported in the most recent version of the compendium of physical activities (Ainsworth et al., 2011). *Sedentary behavior* was measured using a questionnaire based on the principle of the SQUASH. Questions on sedentary behavior concerned sedentary behavior during work, transportation, leisure time (i.e., on work and free days) and resting. Participants were asked how many days a week they spent on sedentary behavior during these activities and how much time on an average day. Sedentary behavior was calculated as a total score of minutes of sitting and lying per week. This questionnaire was designed in another study at the OUNL (van Stralen et al., 2009), but has not been validated or reported up till now. *Chronotype* was measured via reported sleep- and wake-times on work and free days using specific questions from the Munich ChronoType Questionnaire (MCTQ) (Roenneberg et al., 2003). Midsleep (i.e., midpoint of sleep) on free days corrected for sleep debt (MSF_SC_), was used as the measure for chronotype (Roenneberg et al., 2004). This was calculated using the equation: MSF_SC_ = MSF–0.5^*^[SDF–(5^*^SDW + 2^*^SDF)/7]. SDF is sleep duration on free days and SDW is sleep duration on work days. *Sleep quality* was measured with the Pittsburg Sleep Quality Index (PSQI), a well-known and well-validated self-report sleep quality measure (Buysse et al., 1989). The PSQI includes items on subjective sleep quality, sleep latency, sleep duration, habitual sleep efficiency, sleep disturbances, use of sleep medication, and daytime dysfunction. These seven components yield one global score. *Sleep duration* was derived from specific questions from the MCTQ and was included as a polynomial term as an inverted U-shape is present in the relation with cognitive performance (Ferrie et al., 2011; Sternberg et al., 2013). Sleep duration was investigated separately for work and free days. *Breakfast consumption* was measured as eating breakfast every day or not. *Fish consumption* was measured with a questionnaire validated against omega-3 plasma levels (de Groot et al., 2009). *Caffeine consumption* was measured as average daily caffeine consumption calculated from reported coffee, tea and energy drink consumption using average beverage caffeine values reported in the literature (Ruxton, 2008).

#### Covariates

Next to the three BLFs discussed above, five covariates were taken into account:

- alcohol use, as it was positively related to cognitive performance in an observational study conducted among middle-aged adults (average age of 56) (Kalmijn et al., 2002). However, another study among younger middle-aged adults (average age of 43) found no associations between alcohol use and cognitive performance (Caspers et al., 2010).
- educational level (measured on an eight-level scale), as it is a predictor for cognitive performance (Van der Elst et al., 2006a,b,c). In addition, educational level is associated with different lifestyle elements. For example, a higher educational level is related to higher levels of physical activity (Droomers, 2001) and predictive for a healthier nutritional pattern (Darmon and Drewnowski, 2008). The coding represents the following levels: 1, Lower general education; 2, Lower vocational education; 3, Average general education; 4, Average vocational education; 5, Secondary general education; 6, Higher vocational education; 7, Higher general/scientific education; 8, Post-higher/Post-university education.
- age, as it is an important predictor for cognitive performance as well, as cognitive functions decrease with age (Albinet et al., 2012).
- body mass index (BMI; computed from self-reported weight and height using the formula: BMI = weight/height^2^), as it is an important covariate since these three BLFs are associated with BMI. For example, low sleep duration is associated with increased BMI (Taheri et al., 2004).
- computer abilities (measured via a self-developed questionnaire mapping attitude, confidence, and skills toward the use of a computer), as the cognitive tests were conducted via the participants' computer. This could mean that people with lower levels of computer abilities could be slower in responding. This should therefore be only visible in the processing speed measure seeing as the executive measures correct for basic performance (i.e., processes such as motor speed). In the TMT, basis performance was controlled for by subtracting the A-part from the B-part. In the NBT, motor speed plays no role as the number of correctly answered items is the outcome. In the ST no correction for basic performance is included.

### Analyses

Pre-processing and the analyses for descriptives were done in SPSS (version 20; SPSS Inc., Chicago, IL, USA). Path analysis was executed using structural equation modeling (i.e., with only manifest variables) performed in AMOS (version 22.0.0; Arbuckle, 2012). Cross-validation was conducted by randomly splitting the final sample into two subsamples, with an almost equal number of cases in each subsample. One subsample was used as a *testing* sample to develop a model based upon theory and using an *exploratory* mode. The second subsample was used as a *validation* sample to test the developed model in a *confirmatory* mode to check the validity of the developed model.

A covariate model was built, including all covariates mentioned with paths to the dependent measures. Based on the literature, paths from processing speed to both executive variables were drawn. Subsequently, the model fit was evaluated by investigating fit and modification indices using the *testing* subsample. Other relevant parameters were drawn, if necessary. When the fit indices demonstrated proper fit, the statistically non-significant parameters were trimmed (i.e., *P*-value higher than 0.05). The parameter with the highest *P*-value was excluded in a step-by-step mode, re-evaluating the model at each step. This yielded the covariate model. Next, a model was built for the BLFs including all measures mentioned with paths to the dependent measures. The same approach was used as in the development of the covariate model and insignificant parameters were trimmed step-by-step. The last step in the *exploratory* mode was to control for the relevant covariates. This was done by combining both models developed after which the insignificant parameters were trimmed. Finally, this model was tested using the *validation* dataset, in order to evaluate the validity of the final model.

The fit measures reported are the: Normed chi-square (χ*2/df*) (Hair et al., 2014), comparative fit index (CFI: Bentler, 1990), root mean square error of approximation (RMSEA: Browne and Cudeck, 1992) and standardized root mean square residual (SRMR: Byrne, 2010). Proper fit is indicated when: χ*2/df* is less than 3 (Hair et al., 2014), CFI is higher or close to 0.95, RMSEA < 0.06 and SRMR < 0.08 (Hu and Bentler, 1999). Univariate outliers on the three outcome measures were excluded before analyses (i.e., a standardized *Z*-value higher than the absolute value of 3.29 or via visual inspection) in case the distribution was normal.

## Results

### Dataset compilation

The original dataset contained 2842 cases. Participants were excluded if they: (1) did not complete the survey and the cognitive tests (1224 cases); (2) made a remark at the end of the survey that led to exclusion (85 cases); (3) had missing data (346 cases); (4) performed below chance level on the NBT (51 cases); and (5) outliers were excluded as mentioned in the Methods section (five cases). All exclusions mentioned led to the analyses reported below with 1131 people included. This file was then randomly split into a *testing* sample (*N* = 565) and a *validation* sample (*N* = 566) for cross-validation.

To make sure that the exclusions led to a sample that was representative of the initial sample, comparisons were made regarding age, sex, and educational level. No differences were observed regarding age, *t*_(1853)_ = −0.31, *p* = 0.757, as the age of the excluded group with 37.1 years old was almost equal to the included group with 37.3 years old. No differences were observed in terms of sex, χ^2^(1, *N* = 2043) > 0.01, *p* = 0.96, as 38.3% was male in the excluded group, compared with 38.4% in the included group. The apparent sex bias might have influenced the results. Men might outperform women on visual-spatial tasks, which is relevant for the processing speed measurement in the current study. Nevertheless, there is a much greater overlap in the distribution of scores than a difference between them (Weiss et al., 2003). Considering the large sample, this means that analyses can be carried out using the entire sample. In case significant results are found, the sample can be stratified to investigate possible sex differences. Educational level was also equal in both groups, *t*_(2463)_ = −1.69, *p* = 0.091, with 5.82 for the excluded group compared to 5.92 for the included group.

There was no difference in cognitive performance between people who executed the task in one trial or those who interrupted the tasks. For the TMT, the difference between the “one-traillers” (*M* = 20.28; *SD* = 13.03) and the “interrupters” (*M* = 22.53; *SD* = 15.34) was not significant, *t*_(1129)_ = 1.03, *p* = 0.306. For the ST, the difference between the “one-traillers” (*M* = 49.96; *SD* = 7.78) and the “interrupters” (*M* = 54.00; *SD* = 7.60) was not significant, *t*_(1129)_ = 1.86, *p* = 0.063. For the NBT, the difference between the “one-traillers” (*M* = 55.50; *SD* = 5.57) and the “interrupters” (*M* = 56.15; *SD* = 4.96) was not significant, *t*_(1129)_ = 0.79, *p* = 0.431.

### Descriptives

Each model was tested using the method of asymptotically distribution free (ADF) estimation to correct for the non-normality seen in the results since kurtosis values should be around 1 and multivariate kurtosis should not be higher than 5 (Byrne, 2010). Furthermore, the distribution of the outcome measures updating and shifting were not normal. ADF was employed in the analysis because the sample size was more than 10 times the number of freely estimated parameters (Byrne, 2010).

The descriptives are depicted in Table 1. The assumption of no multicollinearity was met following inspection of the correlations (i.e., below 0.8, data not shown, according to Field, 2009). All variables were included in the evaluation of this assumption. Table A1 shows the zero-order correlations between all model variables. As can be seen in Table 1, the standard deviation differs among the variables, imposing a threat to the reliability of the results as homoscedasticity is an important assumption in linear models. Large differences in variances can distort the estimation of the model fit and parameters. The variables were therefore transformed to align the variances prior to the analyses. All descriptives mentioned in Table 1 were reported over the entire dataset before random splitting.

**Table 1:** **Descriptives of all included variables**.

**Variable**	**Mean**	**SD**
Sex (percentage male)	38.4%	–
Age (years)	37.26	10.65
Educational level (ordinal)	5.92	1.37
Body mass index (kg/m^2^)	24.33	4.01
Computer abilities (scale score, higher is better ability)	43.19	5.74
Total weekly alcohol consumption (standard glasses)	3.31	5.31
Physical activity per week (intensity score)^a^	7961.78	4122.48
Physical activity per week (minutes)^b^	2736.53	1160.15
Sedentary behavior per week (minutes)	3040.55	1255.31
Sleep quality score (scale score, higher is lower quality)	6.05	1.85
Sleep duration on work days (hours)^c^	7.89	0.97
Sleep duration on free days (hours)^c^	8.38	1.19
Chronotype (midsleep on free days, corrected for sleep debt)	3.87	1.00
Breakfast consumption (percentage that eats breakfast every day)	77.0%	–
Caffeine consumption (mg/day)	211.38	121.63
Fish consumption (scale score, higher is more omega-3)	10.30	8.10
Processing speed (test score)	50.01	7.78
Shifting (test score)	20.36	13.11
Updating (test score)	55.53	5.55

a Physical activity was calculated as a weekly activity score: an accumulated product score of intensity of the activity multiplied by the minutes spent on the activity;

b To aid interpretation and comparison with the sedentary behavior measure, minutes per week was added for physical activity;

c *Despite a polynomial term is used in the analysis for sleep duration, the original mean and SD are depicted for interpretation purposes*.

### Path analyses

**Figure 1:**
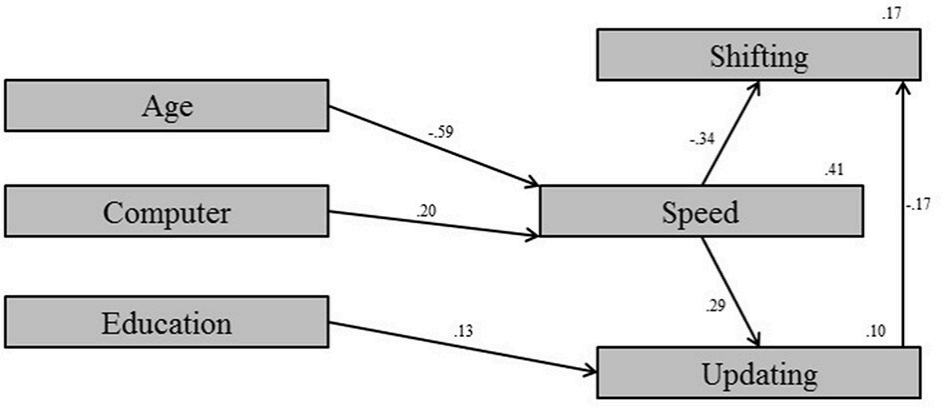
**Model 1: Covariate model with only the relevant parameters**.

**Figure 2:**
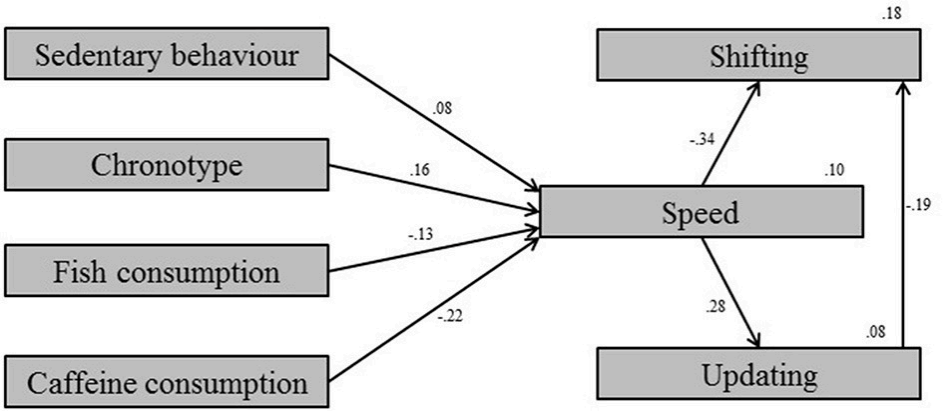
**Model 2: Biological lifestyle factor model with only the relevant parameters**.

**Figure 3:**
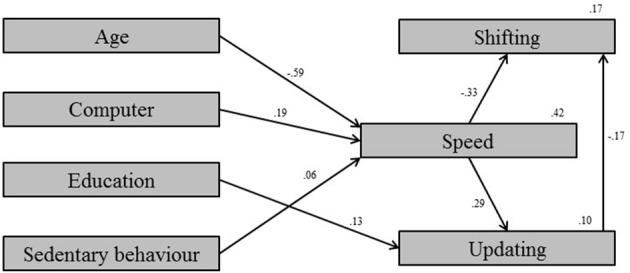
**Model 3: Final trimmed model for the biological lifestyle factor measures after controlling for confounding covariates**.

The *testing* sample was used in the *exploratory* mode for model development. The covariate model which depicted proper fit between the model and the data was built, χ^2^*/df* = 0.79, CFI = 1.00, RMSEA < 0.001, SRMR = 0.018 (Figure 1). Next, the model was built for the BLFs which showed satisfactory fit between the model and the data, χ^2^*/df* = 0.89, CFI = 1.00, RMSEA < 0.001, SRMR = 0.022 (Figure 2). Clearly, four measures within the BLFs show significant relations with processing speed. There were no direct relations from any measure within the BLFs with one of the executive measures. Model 3, which corrected for the covariates, showed good fit between the model and the data, χ^2^*/df* = 0.85, CFI = 1.00, RMSEA < 0.001, SRMR = 0.016 (Figure 3). Model 3 shows that, after controlling for the covariates, only sedentary behavior remains as a significant predictor of processing speed. The relation is positive, meaning that participants who were more sedentary performed better on the processing speed test.

**Figure 4:**
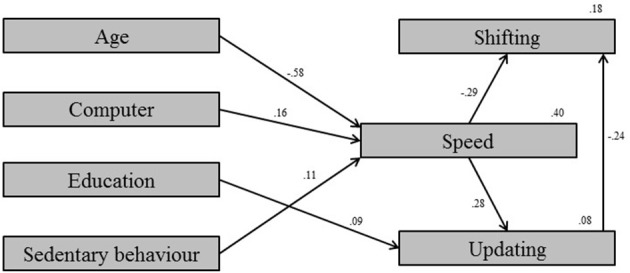
**Validation model: Validation of the developed model (model 3)**.

The *validation* sample was used to test the developed model in the *confirmatory* mode and check the validity of the developed model (i.e., to perform the cross-validation). Validation of the developed model indicated the model was fitting the data properly, χ^2^*/df* = 2.75, CFI = 0.95, RMSEA < 0.056, SRMR = 0.035 (Figure 4). All fit indices are within the limits of proper fit indication. The parameter estimates differ from the estimates in model 3, which used the *testing* sample, but they are comparable (Table 2). In the validation step, exclusion of sedentary behavior indicated that 0.8% of the variance in processing speed was explained by sedentary behavior. This *confirmatory* step in the cross-validation procedure means the model developed in the *exploratory* mode was correct.

**Table 2:** **Estimates for model 3 and the validation model**.

**Estimated parameter**	**Estimate**	***P*-value**
**MODEL 3**		
Regression weight (standardized)		
Sedentary behavior → Speed	0.069	0.045
Computer abilities → Speed	0.194	<0.001
Age → Speed	−0.635	<0.001
Speed → Updating	0.284	<0.001
Education → Updating	0.194	0.002
Speed → Shifting	−0.273	<0.001
Updating → Shifting	−0.142	<0.001
**Squared multiple correlation**		
Speed	0.416	–
Updating	0.097	–
Shifting	0.169	–
**VALIDATION MODEL**		
Regression weight (standardized)		
Sedentary behavior → Speed	0.129	<0.001
Computer abilities → Speed	0.160	<0.001
Age → Speed	−0.652	<0.001
Speed → Updating	0.242	<0.001
Education → Updating	0.119	0.034
Speed → Shifting	−0.219	<0.001
Updating → Shifting	−0.208	<0.001
**Squared multiple correlation**		
Speed	0.404	–
Updating	0.081	–
Shifting	0.176	–

## Discussion

The goal of the present study was to examine whether subjectively measured general habitual behavior on the variables within the BLFs physical activity, sleep, and nutrition were predictive for cognitive performance in young and middle-aged adults. Although a large number of participants needed to be excluded in order to successfully carry out the structural equation modeling, statistical comparison of the included vs. the excluded cases shows no differences in terms of age, sex, and educational level. This means there is no selection bias resulting from these exclusions within the investigated population. In the *exploratory* mode a model was developed using the *testing* sample. This yielded a covariate model (Figure 1), a BLF model (Figure 2), and a final BLF model which was corrected for possible confounding by combining model 1 and model 2 (Figure 3). After correcting for the covariates, sedentary behavior remained as a significant predictor of processing speed. The significant variables chronotype, fish consumption, and caffeine consumption in model 2 (Figure 2) turned out to be insignificant after correcting for the covariates (Figure 3). All other measurements within the BLFs were insignificant. This means that none of the variables measured within the BLFs were predictive for either executive functioning or processing speed, except for sedentary behavior. Sedentary behavior explained a very small proportion of the variance in processing speed. Validation of the developed model via the *confirmatory* step using the *validation* sample showed that the developed model was valid and that the estimates were comparable to the developed model in the *exploratory* mode. This indicated the developed model is generalizable to the population. We will discuss these results per BLF in the light of the literature, after the discussion of the study's limitations.

The current study had several important limitations that we would like to emphasize before discussing the results. First, the study is observational and does not allow for causal inferences although the hypotheses were theory-driven and path analysis assumes directional paths implying causation. Second, the cognitive tests were conducted at the participants' home following the survey which lasted around 45 min. Although participants were instructed to conduct the cognitive tests in a well-rested and active state and without possible distraction, and that they had the liberty to postpone the test and thus separate it from the survey, it could well be that participants did the tests directly after the survey when they were probably fatigued. Alternatively, they might have been distracted by their surroundings (e.g., a pet, child, or partner). Both of these points were tackled by the large data set, however, largely smoothing these possibly confounding factors. Third, survey based research is in many cases not the most reliable type of research because of social desirability, faulty reporting due to memory issues, or question interpretation. Fourth, the time of the day at which the cognitive tests took place was not recorded for the cognitive tests specifically. Despite the large dataset, these limitations may have confounded the results and could impair the interpretation of the results. Fifth, the sensitivity of the cognitive tests might be questioned. The measurement for processing speed clearly shows a relation with a number of predictors and covariates, demonstrating its sensitivity. The executive functions measurements (i.e., updating and shifting) show consistent relations with processing speed, as expected. No relation with the predictors was shown, however, which questions the sensitivity of the measurements. For shifting, the measurement is normally distributed. It could, however, be possible that the sensitivity is too low to reveal relations with the predictors. For updating a ceiling effect is present. Some participants were able to answer all 60 items correct. This is an indication of a possible threat for the sensitivity. On the other hand, updating also shows a relation with education, suggesting the measurement is sensitive enough. Especially considering that education is a large-grained measurement (i.e., an eight-point scale). Last, the investigated sample is quite homogeneous in terms of educational level and all participants are studying for a higher degree. Still, the students investigated are diverse in educational background (i.e., disciplines) and age. Nevertheless, this specific subpopulation might be to homogeneous to reveal possible relations that could be present in the general population of young and middle-aged adults.

Regarding physical activity, the findings were not expected as earlier research showed physical activity to be differentially related to specific cognitive functions (Colcombe and Kramer, 2003; Hillman et al., 2008; Barenberg et al., 2011). However, Barenberg et al. (2011) reported that the function updating was never investigated and that the results on the function shifting were mixed. Hence, these findings are interesting as this is probably the first study that investigated updating and its relation with physical activity. For shifting, the current study added to the body of knowledge. Physical activity shows different relationship with cognitive performance, dependent on the physical activity construct measured (Syväoja et al., 2014). It is therefore of importance to further investigate more specific and objective physical activity constructs. The finding that sedentary behavior was positively predictive for processing speed was also unexpected. This means that participants who were more sedentary performed better on the processing speed test. The measurement of specific types of sedentary behavior is recommended more and more often (Rhodes et al., 2012), as research shows that different types of sedentary behavior (e.g., watching TV vs. working on the computer) are differentially associated with cognitive performance (Kesse-Guyot et al., 2012). For example, Kesse-Guyot et al. (2012) showed TV viewing to be negatively related to cognitive functioning, while computer use was positively related to cognitive functioning. The population investigated in the current study is characterized by fulltime working adult students, who often have very limited time to study due to work and social responsibilities (e.g., children and/or partner). It is therefore very likely that the sedentary time measured here reflects more the sedentary time caused by positive sedentary behaviors (e.g., studying) that stimulates the brain, than negative sedentary behaviors (e.g., TV viewing), since overall sedentary time was measured. We recommend future studies to focus on specific sedentary behaviors to evaluate whether these are differentially related to cognitive performance.

The sleep related measures showed no added value in the prediction of cognitive performance. Although Preckel et al. (2011) showed in their meta-analyses that chronotype was related to cognitive performance, this finding was concluded from research in adolescents/young adults (average age range = 15.19-25.04). In addition, chronotype in this meta-analysis was defined as a two-dimensional construct and cognitive performance was measured using various different aggregated tests. In the current study, participants age range was different and chronotype was defined as a continuous variable (i.e., midsleep on free days corrected for sleep debt; Roenneberg et al., 2004). Cognitive performance was measured for three specific constructs and no overall scores were used. Taken together, these differences can explain the different findings. It could well be that at older ages chronotype is not that strongly related anymore to cognitive performance. In addition, our use of a continuous chronotype measure ensured better estimation of chronotype, so a possible relation with cognitive performance should have stood out if present. It could be that other cognitive functions besides processing speed and executive functioning are associated with chronotype. Second, sleep duration was also not predictive for cognitive performance. Sleep duration was included as a polynomial term (i.e., separate for sleep duration on work and free days) since research shows an inverted U-shaped relation with cognitive performance (Ferrie et al., 2011; Sternberg et al., 2013). Sleep duration not being predictive was unexpected, considering these previous studies. However, the cognitive tests executed in the previous research mentioned included different tests measuring an aggregated set of cognitive functions (Ferrie et al., 2011; Sternberg et al., 2013). This could mean that sleep duration is not associated with processing speed or the executive functions updating and shifting, but that it is associated with combined or other cognitive processes. Third, sleep quality was not shown to be predictive for cognitive performance, partly opposed to the expectations from the literature. Sleep quality not being related to processing speed is in accordance with the literature (Nebes et al., 2009). Sleep quality not being related to executive functioning was unexpected as research suggests a relation (Nebes et al., 2009; Benitez and Gunstad, 2012). However, sleep quality is barely investigated, especially in middle-aged adults. It could be that no relation between sleep quality and executive functioning exists at this age. Considering the limitations of this study, this requires further investigation.

When it comes to nutrition, it was less surprising that breakfast, fish, and caffeine consumption were not predictive for cognitive performance, as these variables were measured on a level of habitual use. Breakfast consumption mostly provides direct effects on cognitive performance (i.e., in the morning, after consumption). Considering that most adults in the current study have full-time jobs and family responsibilities (i.e., children and a partner), they study in the evening hours and will most likely have taken the cognitive tests at night or in the weekend. It is therefore understandable that breakfast consumption did not predict cognitive performance. The relation between fish consumption and cognitive performance in adults is still under debate in the literature (Stonehouse, 2014). From the findings of this study, we conclude that fish consumption does not predict cognitive performance in young and middle-aged adults. Habitual use of caffeine is not predictive for cognitive performance. These findings are in line with previously conducted research. As shown by Killgore et al. (2012), executive functioning is not influenced by caffeine use. In addition, Ruxton (2008) shows in an extensive review that caffeine is expected to have short-term effects on cognitive performance. Since timing of cognitive testing and specific consumption of caffeine use prior to timing was not investigated in the current study, this cannot be evaluated here. Lastly, tolerance effects of caffeine are subject to debate and no agreement is apparent on whether caffeine actually stimulates cognitive performance, or just restores it after tolerance effects appear (Ruxton, 2008). Together, the findings discussed above lead us to conclude that habitual use on these nutrition variables is not predictive for cognitive performance.

The strengths of this study are multiple. The large data set provides a high power related to the findings and decreases the risk of contracting a type-1 error. On the other hand, *explorative* model development increases capitalization on chance. A cross-validation approach was used to control for this. This approach allows us to safely conclude that the model was generalizable to the population. Another strength is that this adult population has rarely been investigated, making these new findings an important starting point for the fields of cognition and individual differences in young and middle-aged adults. Investigating the combination of these three BLFs physical activity, sleep, and nutrition is a new and challenging approach, providing new insights. A major strength is that a number of possible confounders was controlled for, hereby eliminating possible spurious relationships, as shown in the differences between model 2 (Figure 2) and model 3 (Figure 3).

In conclusion, the results presented here indicate that the variables within the BLFs do not predict cognitive performance in young and middle-aged adults. The only exception is sedentary behavior which predicts processing speed, although it predicts only a small proportion of the data. Furthermore, it is unclear which specific sedentary behavior is responsible for this positive relation. Therefore, these results should not promote people to become more sedentary as it is not clear which exact sedentary behaviors are responsible for this effect and research shows sedentary behaviors to be differently associated with cognitive performance (Kesse-Guyot et al., 2012; Rhodes et al., 2012). These results should be interpreted with care and more research is needed to clarify this relation. We suggest future research to investigate the relations between BLFs and their respective relation with, or effect on, cognitive performance as this will create a better understanding of the environmental influences on cognitive performance.

## Author contributions

This study was mainly executed and written by HG, the first author of this research paper. EB supervised HG during his 4-month internship at the Universitat Oberta de Catalunya in Barcelona in 2014. During this time the analyses and writing of the article was done. RD and PK were supervisors of HG during his PhD project and guided him through the process of executing this study and helped him writing the research paper. All co-authors substantially contributed to the work and the subsequent writing of the article.

### Conflict of interest statement

The authors declare that the research was conducted in the absence of any commercial or financial relationships that could be construed as a potential conflict of interest.

## Appendix

**Table A1:** **Correlation table of all variables included in the analyses**.

***N*** = **1131**															
	**Variable**	**1**	**2**	**3**	**4**	**5**	**6**	**7**	**8**	**9**	**10**	**11**	**12**	**13**	**14**
1	Alcohol consumption per week	1	–	–	–	–	–	–	–	–	–	–	–	–	–
2	Educational level	0.079^**^	1	–	–	–	–	–	–	–	–	–	–	–	–
3	Age (in years)	0.174^***^	0.154^***^	1	–	–	–	–	–	–	–	–	–	–	–
4	Body mass index in kg/m^2^	−0.003	−0.024	0.231^***^	1	–	–	–	–	–	–	–	–	–	–
5	Computer abilities (percentage)	−0.007	0.018	−0.132^***^	0.069^*^	1	–	–	–	–	–	–	–	–	–
6	Physical activity (intensity score)	0.084^**^	−0.025	0.198^***^	0.003	−0.018	1	–	–	–	–	–	–	–	−
7	Sedentary behavior	0.103^***^	0.071^*^	−0.004	0.161^***^	0.166^***^	−0.126^***^	1	–	–	–	–	–	–	–
8	Chronotype (MSF_sc_)	0.150^***^	−0.091^**^	−0.282^***^	−0.038	0.132^***^	−0.032	0.153^***^	1	–	–	–	–	–	–
9	Sleep quality	−0.046	0.118^***^	−0.039	0.069^*^	−0.042	−0.075^*^	0.034	0.033	1	–	–	–	–	–
10	Sleep duration polynomial (work)	−0.074	−0.125^***^	−0.071^*^	−0.006	0.022	−0.016	−0.031	0.123^***^	0.060^*^	1	–	–	–	–
11	Sleep duration polynomial (free)	0.044	−0.062^*^	−0.046	−0.040	−0.044	0.059^*^	−0.005	0.102^***^	0.058	0.259^***^	1	–	–	–
12	Breakfast consumption	−0.032	0.159^***^	0.225^***^	−0.016	−0.074^*^	0.041	−0.065^*^	−0.272^***^	−0.091^**^	−0.108^***^	−0.047	1	–	–
13	Caffeine consumption	0.186^***^	0.042	0.392^***^	0.121^***^	−0.035	0.086^**^	0.050	−0.061^*^	0.013	−0.061^*^	−0.027	0.048	1	–
14	Fish consumption	0.112^***^	0.026	0.217^***^	0.031	0.005	0.097^***^	−0.029	0.015	−0.005	−0.043	0.049	0.080^**^	0.082^**^	1

* P < 0.05;

** P < 0.01;

*** *P < 0.001*.
